# Pilot study of factors contributing to canine impaction after secondary alveolar bone grafting in unilateral cleft lip and palate patients

**DOI:** 10.1038/s41598-022-12565-y

**Published:** 2022-05-20

**Authors:** Yoko Tsurumi, Kazuaki Nishimura, Megumi Shimizu, Yoshimichi Imai, Kaoru Igarashi

**Affiliations:** 1 Division of Craniofacial Anomalies, Tohoku University Graduate School of Dentistry, Sendai, Japan; 2 Department of Orthodontics and Speech Therapy for Craniofacial Anomalies, Tohoku University Hospital, 4-1 Seiryo-machi, Aoba-ku, Sendai, Japan; 3 Department of Gerontological and Home Healthcare Nursing, Course of Nursing, Tohoku University Graduate School of Medicine, Sendai, Japan; 4 Department of Plastic and Reconstructive Surgery, Tohoku University Graduate School of Medicine, Sendai, Japan

**Keywords:** Medical research, Risk factors, Diseases, Oral diseases

## Abstract

Alveolar bone grafting (ABG) is broadly performed for cleft lip and palate patients. The rate of canine impaction post-ABG is much higher than the prevalence of canine impaction in non-cleft patients. This pilot study was designed to investigate factors involved in canine eruption failure after ABG and to predict the possibility of canine impaction in unilateral cleft lip and palate (UCLP) patients. This retrospective observational study examined 45 patients with UCLP (mean age 7.9 years) classified into an impacted group (n = 9) and a spontaneously erupted group (n = 36). From 3D images, we measured lateral incisor presence or absence, lateral incisor position on the cleft side, canine position, movement change, cleft volume, and canine and first premolar overlap-area. Multivariate logistic regression analysis using independent variables indicated significant differences in results, selecting highly relevant items. Multivariate analysis indicated a significant association between the overlap-area between the canine and the first premolar at pre-ABG (p = 0.038) and the distance between the cleft side cusp tips of canine and the lateral cleft margin of pre-ABG (p = 0.005). Results suggest that canine impaction is predictable at an early stage in pre-ABG and show the possibility of comprehensive diagnosis of canine impaction using computed tomography.

## Introduction

Treatment of cleft lip and palate patients requires long-term multidisciplinary management from birth to adulthood by the relevant departments^1–3^. Since Boyne et al.^4^ reported alveolar bone grafting (ABG) in 1972, it has become an important procedure for treating patients with cleft lip and palate. The main purpose of ABG is integration of the alveolar and maxillary to stabilize the width of the maxillary bone, ensure continuity, and contour the alveolar bone, the result is likely to be proper distribution of occlusal stress, resulting in more symmetrical and natural development of the maxilla, one of the goals create an eruption tract for permanent teeth, and to support the roots of erupting permanent teeth^5,6^. The rate of canine impaction post-ABG has been reported as 12–35%^7^, which is much higher than the prevalence of canine impaction in non-cleft patients, which is 0.8–3%^7,8^. Impacted teeth are defined as teeth in the submucosa of the oral cavity or in the jaw bone without eruption of all or part of the crown after a certain time of eruption^9^. Common causes of tooth impaction include systemic, local, and genetic factors. Systemic factors include hypoendocrinosis, radiation therapy, and local factors including inadequate root resorption of deciduous teeth, ankylosis, premature deciduousness, excess teeth, abnormal eruption pathways of permanent teeth, insufficient eruption space, trauma, abnormal tooth position, and odontoma. Abnormalities in tooth germ formation caused by genetic factors have also been reported^10^. Causes of canine eruption failure in patients with cleft lip and palate are reported as insufficient space for canine eruption because of the presence of a cleft and the possibility of canine impaction because of the timing of ABG^7^. To date, most studies have used panoramic radiographs, which are insufficient for detailed evaluation of the canine eruption pathway, the cleft morphology, and the positional relation between the canine and the cleft.

Based on results of a computed tomography (CT) study, Oberoi et al.^11^ reported a three-dimensional evaluation of the maxillary canine eruption pathway. They didn’t find any association between congenital absence of lateral incisors and canine root completion, canine eruption failure. Meanwhile, some studies found an association between increase in the rate of canine impaction and agenesis of lateral incisor, with very low certainty of evidence^12^. Some studies mentioned insufficient arch space of CLP patients is proposed to be associated with canine impactions^13^ others mentioned there is no relationship between canine impaction and dental crowding^14^. The angulation of canine eruption before bone graft surgery was considered a risk for impaction by some studies^6,15^. Though other authors have not found this association^16^. The causes of canine eruption failure in cleft lip and palate patients and the factors contributing to a higher proportion of eruption failure than in non-cleft patients remain unclear. Although canine impaction is known to occur more frequently in cleft lip and palate patients, it might be diagnosed several years post-ABG, requiring fenestration or complex orthodontic treatment because of root resorption of adjacent teeth, prolonging the treatment period and increasing the cost of treatment^10,17^, it is important to diagnose the possibility of impaired eruption pre-ABG and post-ABG at an early stage.

This pilot study was designed to elucidate factors involved in canine eruption failure and to predict the possibility of canine impaction in unilateral cleft lip and palate patients using CT images of pre-ABG and post-ABG.

## Results

### Correlation between presence or absence of lateral incisors and buccolingual position on the cleft side and canine impaction

In the erupted group, lateral incisors existed in 23 of 36 (63.9%) cases. Congenital absence was observed in 13 cases (36.1%). By contrast, in the impacted group, lateral incisors existed in 4 of 9 (44.4%) cases. Congenital absence was observed in 5 (55.6%) of the cases. Furthermore, among the 28 cases in which lateral incisors were present, 19 out of 23 cases in the erupted group and 3 out of 4 cases in the impacted group had the lateral incisors located more palatally. No significant difference was found between the presence or absence of lateral incisors and buccolingual position and canine impaction (*p* = 0.68).

### Correlation between the distance between the cleft side canine and the lateral cleft margin at T1 and canine impaction

As shown in Table 1, the cusp tips of the canine were 0 mm < α ≤ 1 mm pre-ABG in one case each in both groups, with no significant difference found. Results show that 4 of 36 cases in the erupted group and 7 of 9 cases in the impacted group had the cusp tips of the canine located at 0 mm < α ≤ 2 mm. Also, 16 of 36 cases in the erupted group and 8 of 9 cases in the impacted group had the cusp tips of the canine located in the range of 0 mm < α ≤ 3 mm. In the impacted group, the cusp tips of the canine were significantly more likely to be located at ≤ 3 mm. The distance between the cleft and the canine was close. No significant difference was found in the distance β between the maximum contour of the pre-ABG cleft side canine and the cleft in any range.

**Table 1:** Distance between the cusp tips of the cleft side canine and the lateral cleft margin at pre-ABG.

α	In	Out	Significance	β	In	Out	Significance
**0 mm < α/β ≤ 1 mm**							
Erupted	1	35	0.36		6	30	1.0
Impacted	1	8			1	8	
**0 mm < α/β ≤ 2 mm**							
Erupted	4	32	0.0002		21	15	0.26
Impacted	7	2			3	6	
**0 mm < α/β ≤ 3 mm**							
Erupted	16	20	0.02		27	9	0.11
Impacted	8	1			4	5	

*α* The shortest distance between the lateral cleft margin and the cleft side cusp tips of a canine, *β* The shortest distance between the lateral cleft margin and the cleft side maximum counter of a canine.

### Correlation between the volume of the cleft and canine impaction

The mean volume of the cleft in the erupted group was 0.98 cm^3^ (maximum 2.11 cm^3^, minimum 0.03 cm^3^). The mean volume of the cleft in the impacted group was 0.97 cm^3^ (maximum 1.10 cm^3^, minimum 0.65 cm^3^). No significant correlation was found between the cleft volume and canine impaction (*p* = 0.98).

### Correlation between canine and first premolar overlap-area and canine impaction in pre-ABG

In all cases, the canine was infra-positioned with the first premolar. The overlap-area was, on average, 20.13 mm^2^ (maximum 46.71 mm^2^, minimum 0 mm^2^) in the erupted group and 29.42 mm^2^ (maximum 43.26 mm^2^, minimum 18.35 mm^2^) in the impacted group. The erupted group was found to have a significantly larger overlap-area in the projection image (*p* = 0.042).

### Correlation between angular changes of canine axis in the non-cleft side and cleft side pre-ABG and post-ABG and canine impaction

As shown in Table 2, on the non-cleft side, no significant difference was found in the angle of the virtual canine axis with respect to each plane between the erupted and impacted groups in either pre-ABG or post-ABG periods. Results show that ∠θ_1_ on the cleft side of the impacted group (59.84°) was significantly smaller than that of the erupted group (71.99°) at T1 (*p* = 0.0001). Additionally, at T2, the ∠θ_1_ of the impacted group (57.80°) was significantly smaller than that of the erupted group (67.89°) (*p* = 0.0001). These findings indicate that, pre-ABG, the impacted group was more horizontally inclined to the XY plane than the erupted group was. This inclination was not improved post-ABG. Also, ∠θ_2_ on the cleft side was 11.32° for the erupted group and 20.49° for the impacted group in T1 (*p* = 0.03), and 13.94° for the erupted group and 20.08° for the impacted group in T2 (*p* = 0.03): both were significantly larger for the impacted group than for the erupted group. These data indicate that the impacted group has more labiolingual tipping than the erupted group pre-ABG. They also indicate that the labiolingual tipping did not improve post-ABG. No significant difference was found in ∠θ_3_ between the erupted and impacted groups (T1: *p* = 0.09, T2: *p* = 0.11), both group canines were not mesial tipping on the cleft sides at T1 and T2.

**Table 2:** Angle and change of canine axis of non-cleft side and cleft side at T1 and T2.

		Non-cleft side			Cleft side		
		Erupted	Impacted	Significance	Erupted	Impacted	Significance
∠θ_1_	T1 (°)	69.3	69.83	0.91	71.99	59.84	0.0001
	SD	14.61	6.8		7.59	4.61	
	T2 (°)	68.74	70.3	0.73	67.89	57.8	0.0001
	SD	13.09	6.72		7.62	3.99	
	Difference (°)	− 0.56	0.46	0.59	− 4.1	− 2.03	0.37
	SD	5.43	3.06		6.51	6.51	
∠θ_2_	T1 (°)	15.88	17.63	0.59	11.32	20.49	0.03
	SD	8.86	8.19		9.37	9.67	
	T2 (°)	16.33	17.59	0.73	13.94	20.08	0.03
	SD	10.02	8.56		7.43	8.15	
	Difference (°)	0.45	− 0.04	0.77	2.61	− 0.41	0.15
	SD	5.02	2.63		6.1	1.91	
∠θ_3_	T1 (°)	− 4.54	− 2.49	0.68	9.54	15.59	0.09
	SD	14.11	8.51		8.44	13.12	
	T2 (°)	− 2.59	0.86	0.47	14.51	20.23	0.11
	SD	13.73	7.44		8.5	12.41	
	Difference (°)	1.95	3.36	0.55	4.97	4.63	0.88
	SD	6.72	4.11		6.46	4.2	

∠θ1, ∠θ2, and ∠θ3 are the minimum angles between the virtual tooth axis and the XY, XZ, and YZ planes, respectively.

### Correlation between movement of the canines of the erupted and impacted groups pre-ABG and post-ABG into the cleft and canine impaction

Pre-ABG, the extension of the virtual canine axis of the cleft side canine passed through the cleft in 5 of 36 cases in the erupted group and in 6 of 9 cases in the impacted group (*p* = 0.006).

Post-ABG, we sought to verify whether the cusp tips of canine had actually moved into the grafted bone region. In the erupted group, it had moved in 6 of the 36 cases. By contrast, in the impacted group, it had entered the grafted bone region in all six of the nine cases in which the pre-ABG extension of the virtual canine axis passed through the cleft (*p* = 0.003). In three cases, it did not move into. In the impacted group at pre-ABG, the extension of the virtual canine axis passed through the cleft significantly more than in the erupted group. It moved significantly into the graft bone area post-ABG.

### Correlation between movement and location of canines on the non-cleft side and cleft side pre-ABG and post-ABG and canine impaction

At pre-ABG, the distance between the origin and the non-cleft side cusp tips of canine in the pre-ABG erupted group (OEa_T1_) was 29.86 mm. The distance between the origin and the cleft side cusp tips of canine (OEb_T1_) was 28.36 mm: a significant difference. The distance between the origin and the non-cleft side cusp tips of canine in the pre-ABG impacted group (OIa_T1_) was 29.94 mm. The distance between the origin and the cleft side canine (OIb_T1_) was 26.76 mm: a significant difference. Post-ABG, the distance between the origin and the non-cleft side cusp tips of canine (OEa_T2_) and the distance between the origin and the cleft side cusp tips of canine (OEb_T2_) in the erupted group were, respectively, 31.16 mm and 29.66 mm. Significant difference was found. The distance between the origin and the non-cleft side cusp tips of canine in the impacted group (OIa_T2_) was 31.71 mm. The distance between the origin and the cleft side cusp tips of canine (OIb_T2_) was 27.52 mm, representing a significant difference. In both the erupted and impacted groups, the distance between the origin and the cusp tips of canine in the pre-ABG was significantly shorter and infra-position in the cleft side than in the non-cleft side. Post-ABG, the distance was significantly shorter and infra-position in the cleft side than in the non-cleft side. No change was found in the infra-position status (Table 3). The difference in the origin–canine distance between the cleft and non-cleft sides in the erupted group (OEaT1–OEbT1) was 1.50 mm in the pre-ABG. The difference in the impacted group (OIaT1–OIbT1) was 3.19 mm, which was not significantly different. The difference between the distance of origin to canine in pre-ABG non-cleft side and cleft side was 1.50 mm in the erupted group (OEa_T1_–OEb_T1_) and 3.19 mm in the impacted group (OIa_T1_–OIb_T1_), representing no significant difference (*p* = 0.053). However, the difference between the distance of the origin to the canine after surgery was 1.51 mm for the erupted group (OEa_T2_–OEb_T2_) and 4.19 mm for the impacted group (OIa_T2_–OIb_T2_), which is a significant difference (Table 4). The difference in origin–canine distances of the impacted group and the erupted group was greater post-ABG than pre-ABG for both the non-cleft side and cleft side.

**Table 3:** Distance between the origin and the canine of non-cleft side and cleft side at T1 and T2.

		Non-cleft side		Cleft side		Significance
		Mean (mm)	SD	Mean (mm)	SD	
Erupted	T1	29.86 (OEa_T1_)	2.61	28.36 (OEb_T1_)	2.68	0.0008
	T2	31.16 (OEa_T2_)	3.44	29.66 (OEb_T2_)	3.21	0.0014
Impacted	T1	29.94 (OIa_T1_)	2.55	26.76 (OIb_T1_)	1.9	0.0001
	T2	31.71 (OIa_T2_)	3.4	27.52 (OIb_T2)_	2.76	0.0001

**Table 4:** Difference between non-cleft side and cleft side origin–canine distances at T1 and T2.

	Erupted		Impacted		Significance
	Difference (mm)	SD	Difference (mm)	SD	
T1	1.50 (OEa_T1_ − OEb_T1_)	2.45	3.19 (OIa_T1_ − OIb_T1_)	0.75	0.0535
T2	1.51 (OEa_T2_ − OEb_T2_)	2.60	4.19 (OIa_T2_ − OIb_T2_)	1.31	0.0002

Further, at the coordinate points evaluation, the erupted group in the pre-ABG was found to have significant differences in all coordinate points (x, y, z) between the non-cleft side, and cleft side canines, compared to the non-cleft side. The cleft side was located, on average, 1.42 mm mesial (positive direction on X-axis), 2.14 mm palatal (positive direction on Y-axis), and 1.08 mm infra-position (positive direction on Z-axis). Post-ABG, there remained significant difference in x and y coordinates between the non-cleft side and cleft side canines. The cleft side remained more mesial and palatal than the non-cleft side. However, the difference in z coordinates, which was significant pre-ABG, was no longer significant. The canine moved in the negative direction of the z-axis post-ABG. In the impacted group, a significant difference was found in the y and z coordinates when comparing the pre-ABG non-cleft side and cleft side. As in the erupted group, the cleft side canine was located 2.38 mm more mesially (positive direction on the y-axis) and 3.06 mm infra-position (positive direction on the z-axis) than the non-cleft side canine. As in the pre-ABG period, significant differences in the y and z coordinates in the post-ABG period were found, with the cleft side canine remaining 1.81 mm palatal (positive direction on the y axis) and 3.90 mm infra-position (positive direction on the z axis) than the non-cleft side canine. In other words, in post-ABG, the impacted group moved less in the negative direction of the z-axis (in the direction of the occlusal plane) than the erupted group. Significant difference was found only in the z-coordinate values for the pre-ABG and post-ABG erupted and impacted groups. The pre-ABG difference between the non-cleft side and cleft side z-coordinate values of the impacted group was 3.06 mm. That value represents a difference of approximately 2.0 mm compared to 1.08 mm for the erupted group. The post-ABG difference between the non-cleft side and cleft side z-coordinate values of the erupted group was 0.46 mm, whereas the difference between the impacted group was 3.90 mm. The difference between the erupted group and the impacted group was about 3.5 mm. Pre-ABG, a difference was found in positions in the z-axis between the non-cleft side and cleft side, but the difference was even greater post-ABG (Tables 5, 6).

**Table 5:** (x, y, z) Coordinates of non-cleft side and cleft side canines in T1 and T2.

		Non-cleft side			Cleft side			Significance
			Mean	SD		Mean	SD	
Erupted	T1	x_a_	14.76	2.72	x_b_	13.34	2.54	0.03
		y_a_	0.3	2.61	y_b_	2.44	2.97	0.0001
		z_a_	− 25.67	3.03	z_b_	− 24.58	2.88	0.0012
	T2	x_a_	14.31	2.96	x_b_	11.63	2.97	0.001
		y_a_	− 0.46	2.71	y_b_	0.9	0.52	0.0001
		z_a_	− 27.36	2.67	z_b_	− 26.89	3.54	0.2
Impacted	T1	x_a_	14.11	2.32	x_b_	12.29	2.7	0.153
		y_a_	0.94	2.67	y_b_	3.32	3.01	0.0008
		z_a_	− 26.18	3.76	z_b_	− 23.11	3.65	0.0001
	T2	x_a_	13.13	2.4	x_b_	10.63	3.91	0.13
		y_a_	0.4	2.98	y_b_	2.21	1.04	0.008
		z_a_	− 28.62	3.53	z_b_	− 24.72	3.74	0.0001

**Table 6:** Difference in (x, y, z) coordinates of the origin–canine distance between the non-cleft side and cleft side in T1 and T2.

		Erupted		Impacted		Significance
		Mean (mm)	SD	Mean (mm)	SD	
T1	x_a−_x_b_	1.42	3.69	1.81	3.45	0.77
	y_a−_y_b_	2.14	1.37	2.38	1.36	0.63
	z_a−_z_b_	1.08	1.84	3.06	0.93	0.0001
T2	x_a−_x_b_	− 2.68	4.56	− 2.42	4.48	0.91
	y_a−_y_b_	− 1.36	1.37	− 1.81	1.55	0.4
	z_a−_z_b_	0.46	2.12	3.90	1.69	0.0001

### Multivariate logistic regression analysis

In Using data from overlap of canine and first premolar in T1, the effect size was calculated to be 0.85 and power (1 − β) was 0.72, and using data on the distance between the apex of the canine and the cleft on the cleft side (0 mm < α ≤ 2 mm), power (1 − β) was calculated to be 0.97. Considering the above results and the limitations of the applicable conditions, a sample size of 45 cases in the univariate analysis seems reasonable.

As shown in Table 7, univariate analysis was applied to all measurement results. Multivariate logistic regression analysis was conducted for three items that were associated significantly with canine impaction: the difference in the origin–canine distance between the non-cleft and cleft sides of T2, overlap-area of the first premolar with the canine on the cleft side, and the distance between the cleft side cusp tips of canine and cleft of T1 (0 mm < α ≤ 2 mm).

**Table 7:** Multivariate logistic regression analysis.

	B	S.E.	*p*	O.R.	95% CI for O.R.	
					Lower	Upper
Difference in the origin–canine distance between the non-cleft and cleft sides of T2	0.52	0.29	0.07	1.68	0.95	2.96
Overlap of canine and first premolar in T1	+ 0.14	0.07	0.038	1.15	1.01	1.33
Distance between the apex of the canine and the cleft on the cleft side (0 mm < α ≤ 2 mm)	− 2.32	0.82	0.005	103.9	4.19	2579.6

*B* estimate, *S.E.* standard error, *p* p-value, *O.R.* odds ratio, *95% CI for O.R.* 95% confidence interval for odds ratio.

Results showed a significant association between overlap-area of the first premolar with the canine on the cleft side and the distance between the cleft side cusp tips of canine and cleft of T1 (0 mm < α ≤ 2 mm), with an odds ratio of 1.15 (*p* = 0.038, 95% CI 1.01–1.33) for the overlap-area and 103.9 (*p* = 0.005, 95% CI 4.19–2579.6) for the distance between the cleft side cusp tips of canine and cleft of T1 (0 mm < α ≤  2 mm).

## Discussion

“Genetic theory” and “guidance theory” are the main causal theories of canine impaction^9,15,18^. Genetic theory includes genetic factors, which cause canine impaction. By contrast, guidance theory is based on the idea that the lateral incisor, which guides eruption of the canine, loses its function for some reason, leading to impaction. In general, congenital absence of maxillary lateral incisors has been reported to occur in 2–16.3% of the general population^18–20^, but in cleft lip and palate patients, the percentage of congenital absence of lateral incisors on the cleft side is reportedly higher: 37.6–58.6%^21–25^. In patients with cleft lip and palate, congenital absence of lateral incisors was found in 72.2% of cases of canine impaction^7^. In patients with cleft, if the lateral incisor is missing, then no other teeth can guide the canine eruption. Only alveolar bone surrounding the cleft is present mesial to the canine. A study of the canine eruption angle change and distance from the cleft using panoramic and posteroanterior cephalogram indicated that effects of the cleft on the canine eruption path decreased concomitantly with increasing distance from the cleft^26^. Therefore, we examined details of the positional relation between the cleft side canine and the cleft in 3D. When the distance between the cusp tip of the canine and the cleft was 3 mm or less, risk of impaction was significantly higher. However, no significant correlation was found between the distance from the maximum contour of the canine to the cleft and impaction. In other words, in patients with cleft, the proximity of the canine to the cleft is an important factor because the lateral cleft margin might play the role of the lateral incisor in guidance theory.

Cleft palate patients have small maxillary^1^, the presence of a cleft can reduce the eruption space and lead to canine impaction^15^. Therefore, to examine the space for canine eruption, we measured the overlap-area between the cleft side canine and its distal adjacent tooth on the projection image in the XY plane. In the impacted group, the overlap-area between the projected image of the cleft side canine and the distal adjacent tooth was significantly larger than that in the erupted group. Greater negative arch length discrepancy (ALD) occurred in the alveolar bone on the cleft side, which predicted insufficient space after eruption. The large overlap-area between the canine and the distal adjacent tooth on pre-ABG CT suggested that this might be a factor affecting the occurrence of canine impaction.

Restoring bone continuity in the cleft area by ABG can resolve negative ALD, eliminate the proximity of the canine to the lateral cleft margin, and provide a canine eruption pathway^1,5,27,28^. Therefore, we evaluated the change in the position of the canine pre-ABG and post-ABG. Comparison of the amounts of canine movement between the erupted and impacted groups shows that the amount of vertical movement was significantly less in the impacted group (Tables 4, 5, 6). That finding implies that cleft-side canines with an infra-positioned cusp tip pre-ABG which do not gain sufficient movement to catch up with the non-cleft side position in post-ABG are likely to become impacted in the future. In the impacted group, the abnormal position and eruption direction of the canine pre-ABG did not improve post-ABG, implicating the canine movement during the eruption as a factor in canine impaction. From a 2D study using panoramic radiographs, it was reported that maxillary canines spontaneously erupted from the grafted bone region after ABG^7,15^. However, earlier reports described the importance of moving the canine to the cleft by orthodontic treatment after canine eruption to prevent resorption of the grafted bone^28,29^, suggesting that the canine does not necessarily erupt at the grafted bone region. Results show that even though the canine appears to have moved into the grafted bone on 2D panoramic radiographs, analyses using 3D images revealed that the canine might not have actually moved into the grafted bone region, but might have passed (anteriorly or posteriorly) through the cleft and erupted as shown in this study.

Because each independent variable was regarded as interrelated, we conducted a multivariate analysis to identify independent variables that significantly affect canine impaction and to provide an index that might better predict future canine impaction. Multivariate logistic regression analysis was applied by selecting as independent variables the three most relevant items showing significant differences in the results. Consequently, the confidence intervals for “distance between the cusp tip of cleft side canine and the cleft in pre-ABG (T1) (0 mm < α ≤ 2 mm)” and “the overlap-area between the canine and first premolar in pre-ABG (T1)” did not include 1, suggesting that they are significant for cleft canine impaction. These items might contribute to early and accurate prediction of future canine impaction by viewing them on 3D images of pre-ABG. It may be possible for clinicians and their patients to consider the high potential for surgical canine traction and to plan treatment to take some measures to prevent loss of eruption space due to delayed canine eruption. This study can be considered a pilot study because it includes survey items that have never been investigated before. These pilot results are promising but they should be interpreted with caution due to the low numbers of patients. There was a limit to increasing the sample size to eliminate the effects of confounding factors such as surgical protocols and surgeons. Considering the disease frequency, it might be difficult to conduct a study with a larger number of patients in the same institution, which was a limitation of this study.

## Conclusion

By confirming the overlap-area of the first premolar with the canine on the cleft side and the distance between the cleft side cusp tips of canine and cleft of T1 on 3D images that cannot be assessed with 2D images, the pilot study results presented herein suggest the possibility of predicting future cleft canine impaction at an earlier stage than conventional diagnostic timing.

## Research objectives and methods

Informed consent has been acquired from all the patients and their parents for CT scanning and analysis of the acquired CT images. This retrospective study was approved by the Institutional Review Board of Tohoku University Graduate School of Dentistry (approval no. 2018-3-006). All experiments were carried out in accordance with the Helsinki declaration and the approved guidelines.

### Subjects

The study population consisted of 45 non-syndromic unilateral cleft lip and palate patients (32 boys and 13 girls) who had CT scans and ABG surgery at our hospital out of 57 patients diagnosed with unilateral cleft lip and palate at our university hospital with the date of first examination from January 2010 to September 2014. Twelve patients were excluded who were transferred to another hospital. The selection of population criteria includes the following: This study groups selection was complete enumeration. The canines have not erupted for several years after ABG, and orthodontic treatment such as surgical fenestration and traction is required, and the impaction of canines have been diagnosed. The mean age at the time of ABG was 7.9 years (median 7.6 years, maximum 10.6 years, minimum 5.9 years). The average interval between pre-ABG and post-ABG CT scans was 9.0 months (median 8.8 months, maximum 14.0 months, minimum 6.3 months). CT images were taken (SOMATOM Definition^®^; Siemens AG, Munich, Germany) with 0.8 mm slice width.

Those with performed fenestration and orthodontic traction of the canine on the cleft side were defined as the impacted group (*n* = 9; right = 4, left = 5). Those with canines spontaneously erupted were defined as the erupted group (*n* = 36; right = 9, left = 27). The mean time to diagnosis of the need for fenestration and traction in the impacted group was 46 months (maximum 84 months, minimum 9 months) after ABG.

## Methods

**Figure 1:**
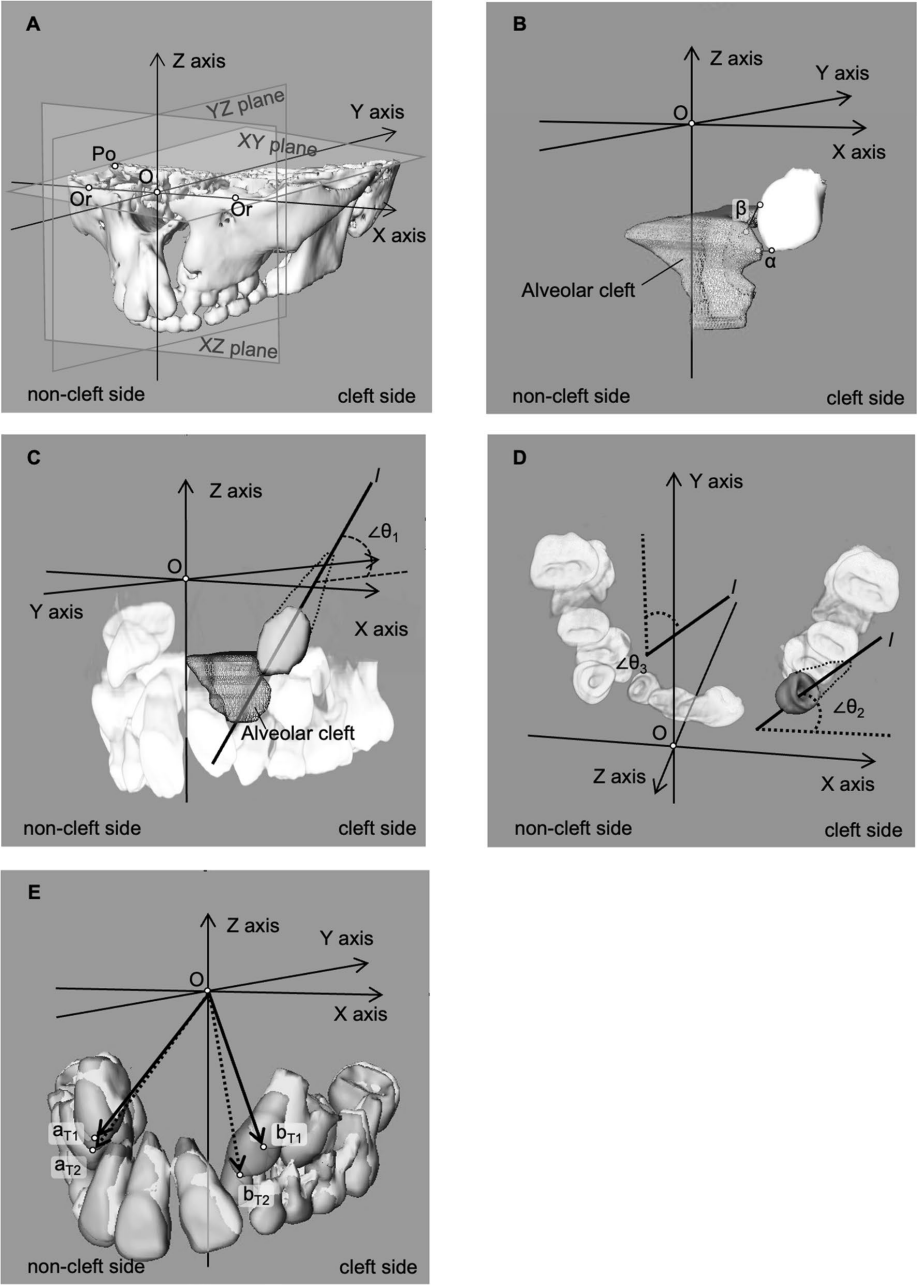
Measurement of canine movement and distance on CT 3D images. **(A)** Arrangement of CT 3D images with 3D coordinates. The midpoint of Or on both sides was set as the origin (O). The plane connecting the three points of the orbitale (Or) on both sides and the porion (Po) on the healthy side mark the XY plane. The plane perpendicular to the XY plane through Or on both sides is the XZ plane. The plane perpendicular to the XY and XZ planes through the origin (O) is the YZ plane. **(B)** The distance between the cleft side canine and the lateral cleft margin of T1. *O* origin, *α* the shortest distance between the lateral cleft margin and the cleft side cusp tips of a canine; β, The shortest distance between the lateral cleft margin and the mesial side of the maximum contour of the cleft side canine. **(C)** The virtual tooth axis of canine was set as *l*. The line passing through the midpoint of the line connecting the mesial and distal sides of the canine crown and the cusp tip of the canine was used as the virtual tooth axis; ∠θ_1_, minimum angle of canine axis to the XY plane. **(D)** ∠θ_2_, minimum angle of canine axis to the XZ plane; ∠θ_3_, minimum angle of canine axis to the YZ plan. **(E)** The (x, y, z) coordinates of the cusp tips of the canine were recorded. The distance was measured on the 3D superimposed image at pre-ABG and post-ABG (pre-ABG, white; post-ABG, gray). a. cusp tips of canine of non-cleft side; b. cusp tips of canine of cleft side; T1, pre-ABG; T2, post-ABG. Oa_T1_: pre-ABG origin-to-canine distance on the non-cleft side, Oa_T2_: post-ABG origin-to-canine distance on the non-cleft side, Ob_T1_: pre-ABG origin-to-canine distance on the cleft side, Ob_T2_: post-ABG origin-to-canine distance on the cleft side.

After conversion to DICOM format, the converted CT data were assembled into three-dimensional (3D) construct images using 3D image analysis software (Amira^®^ ver. 6.4; thermo Fisher Scientific Inc., Massachusetts, USA). The reference plane was set up using the method presented in Fig. 1A, with three points of reference: the left and right orbitals (Or), and the porion (Po) on the non-cleft side. The pre-ABG and post-ABG CT images were overlaid on a voxel basis with image analysis software^30,31^. Positive and negative coordinates are reversed in the cases of left-sided and right-sided clefts. Therefore, all cases of right-sided cleft were inverted after constructing 3D images. All cases were measured as left-sided cleft.

### Presence or absence of lateral incisors and buccolingual position of the lateral incisors on the cleft side

Medical records and pre-ABG CT images confirmed the presence or absence of a lateral incisor and the buccolingual position of the lateral incisors on the cleft side. All permanent teeth between the central incisor and the canine were regarded as lateral incisors irrespective of their morphology or size^21,32^. No missing lateral incisors were identified on the non-cleft side.

### Distance between the cleft side canine and the cleft

The shortest distance between the lateral cleft margin and the cleft side cusp tips of a canine was defined as α. The shortest distance between the lateral cleft margin and the mesial side of the maximum contour of the cleft side canine was defined as β. They were measured using the auto measurement function of software. The following ranges were set: 0 mm < α (β) ≤ 1 mm, 0 mm < α (β) ≤ 2 mm, and 0 mm < α (β) ≤ 3 mm. The numbers in the respective ranges found for the erupted and impacted groups were checked and compared (Fig. 1B).

### Volume of the cleft

After a 3D image of the cleft was constructed using pre-ABG CT data, the extent of the cleft was set on the image by reference to the multi-planar reconstruction (MPR) image. The upper limit of the cleft was set at the inferior margin of the piriform aperture and the lower limit at the alveolar bone crest. It was set anteriorly and posteriorly while checking the alveolar bone on MPR images^33,34^. The volume was found by measuring the area of each MPR image and the number of slices^5^. The respective volumes of the erupted group and the impacted group volume were compared.

### Overlap-area of canine and first premolar in pre-ABG

The crowns of the canine and first premolar were projected onto the XY plane on a 3D image. The respective overlap-area of the erupted and impacted groups were measured and compared.

### Changes in the canine axis angle of pre-ABG and post-ABG on the non-cleft side and cleft side

The root of the canine was not yet complete. Therefore, the line passing through the midpoint of the line connecting the mesial and distal sides of the maximum contour of canine crown and the cusp tip of the canine was used as the virtual tooth axis^35^. The minimum angles (∠θ_1_, ∠θ_2_, and ∠θ_3_) between the virtual tooth axis and the XY, XZ, and YZ planes, as well as the amounts of angle change found for pre-ABG and post-ABG for the erupted and impacted groups were measured and compared (Fig. 1C,D).

### Movement of canine into the grafted bone region of the erupted and impacted groups of pre-ABG and post-ABG

The 3D images of pre-ABG were used to ascertain whether the extension of the virtual tooth axis of the cleft side canine passed through the cleft. In addition, the 3D images for pre-ABG and post-ABG were superimposed to verify whether the cusp tips of canine had moved into the grafted bone in post-ABG.

### Movement and location of canines on the non-cleft side and cleft side pre-ABG and post-ABG

We assigned variables to various parameters. Pre-ABG was set as T1. Post-ABG was T2. The apex of the non-cleft side canine was *a.* The apex of the cleft canine was *b*. The erupted group was *E*. The impacted group was *I.* Apex coordinates of the non-cleft side canine (x_*a*,_ y_*a*_, z_*a*_) and the apex coordinates of the cleft side canine (x_*b*_, y_*b*,_ z_*b*_) were recorded. The following items were measured and compared (Fig. 1E).

For the non-cleft and cleft side, we compared the following: distance from the origin to the cusp tips of canine in the pre-ABG erupted group (OE_*a*T1_ and OE_*b*T1_), distance from the origin to the cusp tips of canine in the post-ABG erupted group (OE_*a*T2_ and OE_*b*T2_), distance from the origin to the cusp tips of canine in the pre-ABG impacted group (OI_*aT1*_ and OI_*bT1*_), and distance from the origin to the cusp tips of canine in the post-ABG impacted group (OI_*aT2*_ and OI_*bT2*_). Differences in the distance from the origin to the cusp tips of canine for the non-cleft and cleft sides at T1 and T2 for the erupted and impacted groups were also compared.

The coordinates of non-cleft side cusp tips of canines (x_*a*_, y_*a*_, z_*a*_) and the coordinates of cleft side cusp tips of canines (x_*b*_, y_*b*_, z_*b*_) were compared. Differences in coordinates of non-cleft side and cleft side canines at T1 and T2 of the erupted group and impacted group were compared.

### Statistical analysis

Descriptive statistics were calculated for each item. Canine eruption or impaction was set as the dependent variable. Since this pilot study was a retrospective study of rare cases, the number of samples that can be collected was limited. The sample numbers were small for the multivariate analysis using all independent variables examined, an element of coincidence could not be eliminated, and the results of the analysis might be less reliable. Therefore, after univariate analysis was applied to all measurement outcome, multivariate logistic regression analysis was applied only to three independent variables significantly associated with canine impaction. Power for univariate analyses was confirmed by post hoc power analysis with G*power (Heinrich Heine University Dusseldorf, Germany) , and univariate power was calculated to be generally reasonable. Univariate analysis was performed using the following statistical analyses: Fisher’s exact probability test was used for the categorical data, and two-sided t-test was adapted: the presence or absence of a lateral incisor on the cleft side, distance between the cleft side canine and the cleft at pre-ABG, extension of the virtual tooth axis of the cleft canine at pre-ABG passing through the cleft, and movement of the canine apex into the grafted bone region at post-ABG. Numerical data (cleft volume, overlap-area of the canine and first premolar on the cleft side, angular changes in the non-cleft side and cleft side canine axis pre-ABG and post-ABG, difference of origin–canine distance shown in Table 4 and difference of canine movement pre-ABG and post-ABG shown in Table 6 on the non-cleft side and cleft side) were tested for equality of variance, followed by Student’s *t*-test. When equality of variance was rejected, Welch's test was performed. In the numerical data, a paired t-test was used for the comparison of the non-cleft and cleft origin-to-canine distance (Table 3) and canine movement (Table 5) between the non-cleft and cleft sides before and after ABG. Significance was inferred for results showing 5% for both sides. All statistical analyses were conducted using software (JMP^®^ ver.14.2; SAS Institute Inc., North Carolina, USA).

## Data Availability

All data are available in the main text. The data sets generated during and/or analyzed during the current study are available from the corresponding author on reasonable request.
